# Multimodal approach to the treatment of patients with radioiodine refractory differentiated thyroid cancer and metastases to the central nervous system

**DOI:** 10.1002/cam4.4901

**Published:** 2022-10-06

**Authors:** Teresa Alonso‐Gordoa

**Affiliations:** ^1^ Medical Oncology Department Hospital Universitario Ramón y Cajal Madrid Spain; ^2^ Instituto de Investigación Biomédica Ramón y Cajal (IRICYS) Madrid Spain

**Keywords:** central nervous system, differentiated thyroid cancer, radiosurgery, radiotherapy, surgery, tyrosine kinase inhibitors

## Abstract

The diagnosis of central nervous system metastases in patients with radioiodine refractory differentiated thyroid cancer is a late and rare event that occurs in less than 1% of patients. Definitive conclusions on the overall clinical management cannot be drawn due to the limited number of patients included in retrospective series or post hoc analysis from clinical trials. However, most data show a trend to an increased benefit from a multimodal approach. Local treatment based on surgical and/or radiation techniques is highly encouraged for symptom control and to reduce tumor burden in this location despite a high risk of clinical complications. In addition, systemic treatment with novel tyrosine kinase inhibitors has demonstrated activity in this subgroup of patients, improving an otherwise unfavorable prognosis.

## Graphical abstract

Central nervous system metastases in patients with radioiodine refractory differentiated thyroid cancer is rare, so the definitive clinical management remains uncertain. However, there is a trend to an increased benefit from a multimodal approach of local surgical and/or radiation techniques and systemic treatment with novel tyrosine kinase inhibitors. The article presents a review of the current data available for this treatment approach.

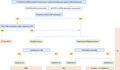

## 1 INTRODUCTION

The presence of metastatic disease in differentiated thyroid cancer (DTC) occurs in approximately 20% of diagnosed patients, either locally or distant. The most common locations for distant metastases in DTC are the lungs, followed by bone.^1^ However, the identification of metastatic disease in the central nervous system (CNS) is rare and is estimated to occur in less than 1% of patients.^1^ In addition to being considered a rare site of metastasis, the CNS is associated with the end stage of the disease and, therefore, with an unfavorable prognosis.

The CNS is considered a sanctuary organ with particular characteristics due to the clinical presentation that metastatic lesions produce in this location, the differentiated treatment required by lesions in this site, and the negative prognostic impact on patient survival.^1^ Additionally, patients with CNS metastases have not been included or have been under‐represented in clinical trials. Consequently, the antitumor activity of the different treatment options has not been well addressed in this particular location.

Overall, there is an unmet clinical need for evidence‐based recommendations on the optimal management of patients with advanced DTC. This article presents a comprehensive review of the current data available for the treatment of this particular clinical scenario.

## 2 HISTORICAL PERSPECTIVE

For many years, the treatment of patients with advanced DTC was based on surgery, radiotherapy, and radioiodine, because low antitumor response was expected with classic chemotherapy in refractory patients. Therefore, the appearance of effective tyrosine kinase inhibitors (TKIs) has modified the current treatment landscape for patients with advanced radioiodine refractory (RAI) DTC.^2^ However, as previously mentioned, patients with CNS metastases are often not selected for clinical trials. The SELECT trial, which demonstrated the benefit of lenvatinib compared with placebo in RAI DTC patients, enrolled patients with known brain metastases, but they needed to be asymptomatic or to have been treated and remained stable with whole brain radiotherapy (WBR), stereotactic radiosurgery (SRS), or complete surgical resection without concomitant corticosteroid treatment.^2^ However, the low incidence of this tumor spread limits the scientific evidence for definitive conclusions in the overall management of this complication. In fact, most data come from retrospective case series.^3^ Nevertheless, most of the evidence highlights the benefit of metastases‐directed therapy even if the prognosis is unfavorable.^3^ In addition to local treatment, the effectiveness of systemic therapies is a matter of concern, particularly in relation to the blood–brain barrier for drug penetration. For this reason, preclinical studies have been carried out with new‐generation TKIs, such as cabozantinib or lenvatinib.^4^, ^5^ Recently approved drugs with selective RET or NTRK inhibitors have shown promising preclinical and clinical activity in metastatic CNS disease. For example, selpercatinib has shown activity in murine models of brain‐implanted tumors that exhibit RET fusion.^6^ However, few patients can benefit from these drugs because *RET* rearrangements have been described in approximately 10% to 20% of patients with papillary thyroid carcinoma and *NTRK* fusions in 5% to 25% of patients with DTC.^7^

## 3 CURRENT SITUATION

The worse survival of patients with radioiodine refractory DTC (RR‐DTC) is related to symptomatic multifocal CNS lesions in the context of a high burden of extracerebral disease.^1^ Fortunately, in recent years, the inclusion of TKIs (mainly lenvatinib and sorafenib) in the therapeutic armamentarium of RR‐DTC has helped to improve the survival of these patients.^2^, ^8^ In particular, lenvatinib activity has been analyzed in patients with brain metastases. Post hoc analysis from the SELECT trial showed encouraging data from patients responding to lenvatinib treatment.^9^ Overall, the median duration of response was 30.0 months (95% confidence interval [CI]: 18.4–36.7). Among five patients with brain metastases, the median duration of response to lenvatinib was 9.3 months (95% CI: 0.9–13.8).^9^ A high burden of disease, liver metastases, and brain metastases were related to a shorter response. A retrospective analysis from Australia reported a median overall survival (mOS) with lenvatinib of 12 months (range: 1–22 months) in the subgroup of patients harboring brain metastases.^10^

Increasing experience with new surgical and radiation techniques, as well as image‐guided neuro‐navigation systems, have helped expand and increase the antitumor response rates in this metastatic location. Scientific evidence for this locoregional approach comes from different retrospective series. The role of postoperative radiotherapy was assessed in a series of 16 patients with thyroid cancer and brain metastases that showed a numerically superior median survival for patients receiving postoperative radiotherapy compared to surgery alone (15 months vs. 27 months; *p* = 0.390).^11^ Additionally, for patients who are not candidates for a surgical approach, Bernad et al. reviewed the role of SRS in 15 patients, four of whom received SRS in the surgical bed.^12^ The median number of lesions treated was 1.5 (range 1–9). Although not statistically significant, a longer mOS was identified in patients receiving this treatment than for those who did not receive SRS (37.4 months vs. 12.3 months; *p* = 0.29). Another study also supported the role of SRS in patients with brain metastases.^13^

Finally, the arrival of new‐generation TKIs such as selective RET and TRK inhibitors for molecularly selected patients have improved the antitumor response in this particular group of patients.^14^, ^15^ Although only a small group of patients harbors these genetic alterations, the magnitude of benefit achieved with these molecularly selected drugs highlights the need to perform a molecular assessment in the RR‐DTC population.

Thus, the potential beneficial role of multimodal treatment based on surgery/SRS and systemic therapy with a TKI has been endorsed.^16^ In a retrospective series of 24 patients, the mOS of patients undergoing SRS compared with those not treated with SRS was 52.5 months versus 6.7 months (*p* = 0.11). For patients undergoing surgery, the mOS was 27.3 months versus 6.8 months for those who did not receive surgery (*p* = 0.15). In addition, for the 12 patients treated with a TKI, the mOS was 27.2 months compared with 4.7 months for those who were not given a TKI (*p* < 0.05). In contrast, the mOS was similar for patients treated or not treated with WBR (21.3 months versus 19.1 months). Nevertheless, when interpreting these data and translating them into clinical practice, it is important to take into account selection biases in retrospective series, as well as the inclusion of different histology subtypes other than differentiated tumors.

Retrospective studies have tried to optimize treatment decision‐making based on key clinical variables [Table 1, ^1^, ^3^, ^11^, ^16^, ^17^, ^18^, ^19^, ^20^]. Some prognostic factors have been suggested to be related to improved survival, such as age ≤ 60 years, Eastern Cooperative Oncology Group (ECOG) Performance Status (PS) ≤2, up to three brain metastatic lesions, and the absence of extracranial metastasis prior to brain metastases.^3^ Patients without risk factors exceed 30 months of survival. In contrast, patients with more unfavorable prognostic factors had a shorter survival below 2 months. ECOG PS was found to be an independent prognostic factor at the time of brain metastases appearance. mOS was 27 months for patients with an ECOG PS <2 versus 3 months for patients with ECOG PS ≥2 (*p* = 0.0009).^11^ No clear differences have been identified according to histological subtypes.^10^ However, the number of patients are small.

**TABLE 1:** Series of patients with advanced thyroid cancer and central nervous system metastases

Study	*N*	Histological subtype	Extra‐CNS metastases	Surgery	SRS	WBR	mOS	Potential prognostic factors^a^
Gomes‐Lima et al. 2019^16^	24	P: 15 F: 7 H: 1	23	10 (2 surgery + SRS) (1 Surgery + SRS + WBR) (5 Surgery + WBR)	8 (2 surgery + SRS) (1 WBR + SRS) (1 Surgery + SRS + WBR)	10 (1 WBR + SRS) (1surgery + SRS + WBR) (3 WBR + surgery)	19 months	NR
Osborne et al. 2019^17^	79	P: 42 PD: 29 F: 4 H: 3	Yes (not specified)	27		63	18 months	Time from DTC diagnosis and CNS metastases development (<3 years)
								Craniectomy
								≤3 CNS lesions
Hong et al. 2018^18^	16	P: 7 F: 4 H: 1 PD: 2 A: 1 M: 1	16	12 (9 surgery + RT)		4	CSS at 1 year: 56.3%	Skull metastases
								Surgical resection of brain lesion(s)
Slutzky‐Shraga et al. 2018^19^	10	P: 6 PD: 1 F: 1 T: 1 I: 1	10	4 (3 surgery + RT) (1 surgery + SRS)	4	1	15 months	NR
Choi et al. 2016^3^	37	P: 32 F: 3 PD: 2	—	3 + 4 (4 Surgery + WBR)	8 + 3 (3 WBR + SRS)	19	8.8 months	Age ≤ 60 years
								ECOG PS ≤2
								≤3 brain metastatic lesions
								Absence of extracranial metastasis
Simoes‐Pereira et al. 2016^1^	27	P: 15 F: 2 H: 2 PD: 5 M: 3	21	5	5 (4 SRS + WBR)	17	5 months	Surgical resection of brain lesion(s)
Saito et al. 2016^20^	24	P: 18 F: 7	—	5	6	4	CSS at 1 year: 28%	Treatment for brain metastasis
Henriques de Figueiredo et al. 2014^11^	21	P:12 F: 5 PD: 4	17	10	2	15	7.1 months	ECOG PS <2
								Surgical resection or SRS of brain lesion(s)
Bernad et al. 2010^12^	23	P: 9 H: 2 M: 1	—	2 + 10 (6 surgery + SRS) (1 Surgery + SRS + WBR) (3 Surgery + WBR)	7 + 8 (6 surgery + SRS) (1 WBR + SRS) (1 Surgery + SRS + WBR)	3 + 5 (1 WBR + SRS) (1surgery + SRS + WBR) (3 WBR + surgery)	20.8 months	KPS > 70%

Abbreviations: A, anaplastic carcinoma; CNS, central nervous system; CSS, cancer‐specific survival; DTC, differentiated thyroid cancer; ECOG PS, Eastern Cooperative Oncology Group Performance Status; F, follicular carcinoma; H, Hürthle cell carcinoma; I, insular carcinoma; KPS, Karnofsky Performance Status; M, medullary carcinoma; mOS: median overall survival; NR, not reported; P, papillary carcinoma; PD, poorly differentiated carcinoma; RT, radiotherapy; SRS, stereotactic radiosurgery; T, tall cell; WBR, whole brain radiotherapy.

^a^ This column includes only significant prognostic factors identified in multivariable analysis.

## 4 TREATMENT RECOMMENDATIONS

**FIGURE 1:**
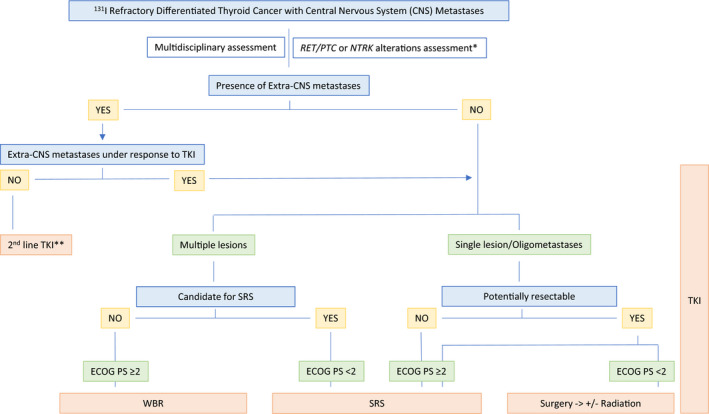
A proposed algorithm for patients with radioiodine refractory differentiated thyroid cancer and metastases to the central nervous system. *Consider primarily starting selective RET or TRK inhibitors if clinically feasible according to the regulatory authorities (i.e., European Medicines Agency approval of these targeted drugs after first‐line treatment). **If clinically indicated. Consider WBR or SRS according to ECOG PS. CNS, central nervous system; ECOG PS, Eastern Cooperative Oncology Group Performance Status; SRS, stereotactic radiosurgery; TKI, tyrosine kinase inhibitor; WBR, whole‐brain radiotherapy.

In general, a local approach to disease in the CNS is recommended regardless of radioiodine avidity. In the radioiodine refractory setting, initiation of systemic TKI treatment is highly encouraged in addition to local therapy.^16^ A therapeutic algorithm for patients with RR‐DTC and CNS metastases is proposed in Figure 1.

On the one hand, if the lesion(s) (a single or limited number of brain metastases) are resectable, the patient should be offered surgical treatment.^3^ The additional benefit from subsequent radiotherapy after complete surgical resection remains controversial. On the other hand, if the lesions are not resectable or the patient is not amenable to a surgical approach, other options such as SRS or WBR should be offered. For example, WBR would be considered for patients with multiple metastases in the CNS, with disseminated disease, who are not candidates for a local/locoregional approach and with a short life expectancy. As previously shown, in this context, TKI treatment should be considered in light of radiological progression in a new location related to poor survival and at high risk of serious complications. In addition, as reported in retrospective studies, most patients have extra‐CNS metastases.^16^ More controversial is the case of a single, completely resected CNS lesion where no other evidence of disease is macroscopically identifiable. In those patients, variables such as the time to CNS lesion development, success of local therapy, histological subtype (i.e., aggressive variants), comorbidities, and ECOG PS should be taken into account. During TKI treatment in these patients, it will be important to monitor the risk of bleeding and adverse cardiovascular events.^1^

For those patients already receiving TKIs, concomitant treatment with the local CNS approach should be avoided due to the involvement of antiangiogenic agents in vascular integrity and wound healing. In this sense, extrapolated from the guidance given in a phase III study involving patients with advanced renal cell carcinoma receiving lenvatinib, we recommend temporarily interrupting the TKI 7 days before any major procedure, such as surgery, and restarting it 7 days afterwards once proper healing is confirmed.^21^ For patients receiving radiotherapy, based on our experience, we recommend discontinuing lenvatinib at least 24 hours before radiation. Once all doses have been administered, lenvatinib can be restarted 24 hours later. We also recommend closely monitoring any related toxicity during the first few days of systemic treatment reintroduction and local approach ending.

Recently, next‐generation sequencing analysis has made it possible to identify potential therapeutic targets such as rearrangement in RET/PTC or alterations in *NTRK*. These patients are amenable to treatment with RET or TRK inhibitors. Early trials with these drugs and other directed therapies such as BRAF inhibitors, show promising results in patients with CNS disease.^14^, ^15^, ^22^ These patients are likely to receive targeted systemic treatment initially in order to delay radiation‐ or surgical‐related complications.

## 5 CLINICAL CASE WITH DISCUSSION

A 68‐year‐old woman presented with a metastatic poorly DTC in September 2016. The patient underwent total thyroidectomy (pT3bN1a stage) and received one course of radioiodine (^131^I) 150 mCi, with a reduction in thyroglobulin levels of >50% and tumor growth control of lung metastatic bilateral lesions (up to 2 cm diameter).

After 18 months, the patient presented with biochemical progression, so a second course of ^131^I 150 mCi was administered. Radioiodine refractory disease was diagnosed 8 months later after new biochemical and radiological lung progression was confirmed. 18F‐fluoro‐2‐deoxy‐D‐glucose positron emission tomography‐computed tomography (CT) identified lung lesions with high metabolic avidity.

**FIGURE 2:**
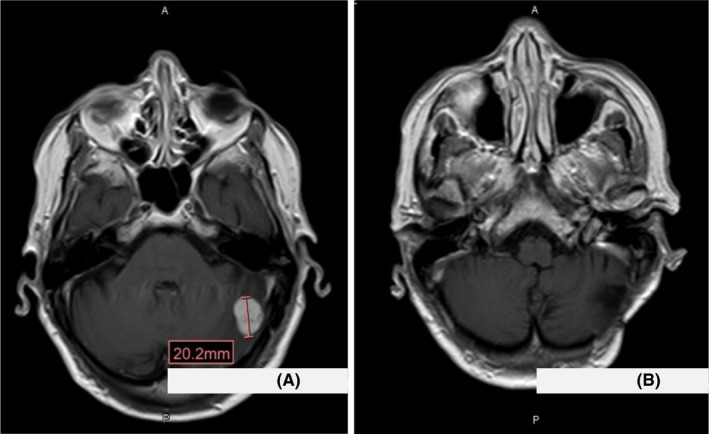
Cranial magnetic resonance images. (A) Shows the metastatic lesion in the left cerebellar hemisphere (January 2019). (B) Shows the absence of residual disease in the surgical bed during follow‐up (July 2019).

In November 2018, the patient started treatment with lenvatinib 24 mg/day but, 2 months later, she presented to the emergency room with headache, gait disturbance, and loss of balance. The cranial CT showed a metastatic lesion of 2.2 cm in the left cerebellar hemisphere with vasogenic edema, and cranial magnetic resonance imaging confirmed this lesion (Figure 2A). At this point, a partial response of lung lesions to lenvatinib was confirmed in the body CT.

In the multidisciplinary committee, surgical resection of the lesion was considered and was carried out in January 2019 after lenvatinib interruption 1 week previously. After recovery from surgery, the patient completed postoperative radiotherapy to the surgical bed. The day after finishing the local treatment and confirmation of the patient's clinical recovery, lenvatinib was reintroduced (Figure 2B).

After 30 months free of disease progression, the patient presented with growth of the metastatic lung lesions, significant increase of mediastinal lymph node lesions, and metastatic pleural effusion. A next‐generation sequencing assessment of tumor tissue identified a CCDC6‐RET rearrangement, so the patient started treatment with a selective RET inhibitor. There is currently a significant reduction of all metastatic lesions, pleural effusion resolution, and no recurrence of brain metastasis.

The presence of metastatic disease in the brain is a rare and late event in the natural history of RR‐DTC. The available evidence from retrospective series or post hoc analysis underscores the benefit of a multimodal approach that includes local treatment with surgery or radiation, if the clinical situation allows, with the addition of a systemic treatment with a TKI. Owing to the arrival of new‐generation TKIs, these patients are benefiting from a trend for improved survival outcomes.

## 6 CONCLUSION

The optimal management of patients with DTC and CNS metastasis requires a multimodal approach combining local treatment and systemic TKI whenever possible. Novel‐targeted agents may change, in the near future, the current management of this particular scenario due to a potentially greater intracranial antitumor activity.

## FUNDING INFORMATION

The author received an honorarium payment from Eisai Farmacéutica SA in line with ICMJE guidelines.

## CONFLICT OF INTEREST

Dr Teresa Alonso‐Gordoa has received research funding, honoraria, and non‐financial or other support from IPSEN, Adacap, Pfizer, Sanofi, EISAI, Lilly, Bayer, Janssen, BMS, Astellas, Novartis, Roche and Merck.

## Data Availability

Data sharing is not applicable to this article as no new data were created or analyzed in this study.

## References

[1] Simoes‐Pereira J , Macedo D , Bugalho MJ . Clinical outcomes of a cohort of patients with central nervous system metastases from thyroid cancer. Endocr Connect. 2016;5:82‐88. 27856495 10.1530/EC-16-0049 PMC5148797

[2] Schlumberger M , Tahara M , Wirth LJ , et al. Lenvatinib versus placebo in radioiodine‐refractory thyroid cancer. N Engl J Med. 2015;372(7):621‐630. 25671254 10.1056/NEJMoa1406470

[3] Choi J , Kim JW , Keum YS , Lee IJ . The largest known survival analysis of patients with brain metastasis from thyroid cancer based on prognostic groups. PLoS One. 2016;11(4):e0154739. 27128487 10.1371/journal.pone.0154739 PMC4851375

[4] Zhang Y , Guessous F , Kofman A , Schiff D , Abounader R . XL‐184, a MET, VEGFR‐2 and RET kinase inhibitor for the treatment of thyroid cancer, glioblastoma multiforme and NSCLC. IDrugs. 2010;13(2):112‐121. 20127563 PMC3268517

[5] Wang R , Yamada T , Arai S , et al. Distribution and activity of lenvatinib in brain tumor models of human anaplastic thyroid cancer cells in severe combined immune deficient mice. Mol Cancer Ther. 2019;18:947‐956. 30926637 10.1158/1535-7163.MCT-18-0695

[6] Subbiah V , Velcheti V , Tuch BB , et al. Selective RET kinase inhibition for patients with RET‐altered cancers. Ann Oncol. 2018;29(8):1869‐1876. 29912274 10.1093/annonc/mdy137 PMC6096733

[7] Román Gil M , Pozas J , Molina‐Cerrillo J , et al. Current and future role of tyrosine kinases inhibition in thyroid cancer: from biology to therapy. Int J Mol Sci. 2020;21(14):4951. 10.3390/ijms21144951 PMC7403957 32668761

[8] Brose M , Nutting CM , Jarzab B , et al. Sorafenib in locally advanced or metastatic, radioactive iodine‐refractory, differentiated thyroid cancer: a randomized, double‐blind, phase 3 trial. Lancet. 2014;384(9940):319‐328. 24768112 10.1016/S0140-6736(14)60421-9 PMC4366116

[9] Gianoukakis AG , Dutcus CE , Batty N , Guo M , Baig M . Prolonged duration of response in lenvatinib responders with thyroid cancer. Endocr Relat Cancer. 2018;25(6):699‐704. 29752332 10.1530/ERC-18-0049 PMC5958278

[10] Rendl G , Sipos B , Becherer A , et al. Real‐world data for Lenvatinib in radioiodine‐refractory differentiated thyroid cancer (RELEVANT): a retrospective multicentric analysis of clinical practice in Austria. Int J Endocrinol. 2020;2020:8834148. doi:10.1155/2020/8834148 33312196 PMC7719524

[11] Henriques de Figueiredo B , Godbert Y , Soubeyran I , et al. Brain metastases from thyroid carcinoma: a retrospective study of 21 patients. Thyroid. 2014;24:270‐276. 23734630 10.1089/thy.2013.0061

[12] Bernad DM , Sperduto PW , Souhami L , Jensen AW , Roberge D . Stereotactic radiosurgery in the management of brain metastases from primary thyroid cancers. J Neurooncol. 2010;98:249‐252. 20376550 10.1007/s11060-010-0175-z

[13] Kim IY , Kondziolka D , Niranjan A . Gamma knife radio‐surgery for metastatic brain tumors. J Neurooncol. 2009;93(3):355‐359. 19139821 10.1007/s11060-008-9783-2

[14] Andreev‐Drakhlin A , Cabanillas M , Amini B , Subbiah V . Systemic and CNS activity of selective RET inhibition with Selpercatinib (LOXO‐292) in a patient with RET‐mutant medullary thyroid cancer with extensive CNS metastases. JCO precis Oncologia 2020;4:PO.20.00096. doi:10.1200/po.20.00096 PMC7608524 33154983

[15] Patel JY , Farago A , Hong D , et al. Activity of larotrectinib in TRK fusion cancer patients with CNS metastases. Presented at SNO Virtual Meeting 19‐21 November 2020. CTNI‐04. URL: https://academic.oup.com/neuro‐oncology/article/22/Supplement_2/ii41/5960557. doi: 10.1093/neuonc/noaa215.171

[16] Gomes‐Lima CJ , Wu D , Rao SN , et al. Brain metastases from differentiated thyroid carcinoma: prevalence, current therapies, and outcomes. J Endocr Soc. 2018;3(2):359‐371. 30706042 10.1210/js.2018-00241 PMC6348752

[17] Osborne JR , Kondraciuk JD , Rice SL , et al. Thyroid cancer brain metastasis: survival and genomic characteristics of a large tertiary care cohort. Clin Nucl Med. 2019;44(7):544‐549. 31107749 10.1097/RLU.0000000000002618 PMC6546545

[18] Hong YW , Lin JD , Yu MC , Hsu CC , Lin YS . Outcomes and prognostic factors in thyroid cancer patients with cranial metastases: a retrospective cohort study of 4,683 patients. Int J Surg. 2018;55:182‐187. 29883619 10.1016/j.ijsu.2018.06.001

[19] Slutzky‐Shraga I , Gorshtein A , Popovitzer A , et al. Clinical characteristics and disease outcome of patients with non‐medullary thyroid cancer and brain metastases. Oncol Lett. 2018;15(1):672‐676. 29387239 10.3892/ol.2017.7325 PMC5768058

[20] Saito F , Uruno T , Shibuya H , et al. Prognosis after brain metastasis from differentiated thyroid carcinoma. World J Surg. 2016;40(3):574‐581. 26762631 10.1007/s00268-016-3405-5

[21] Motzer R , Alekseev B , Rha SY , et al. Lenvatinib plus pembrolizumab or everolimus for advanced renal cell carcinoma. N Engl J Med 2021. doi: 10.1056/NEJMoa2035716. Epub ahead of print 33616314

[22] Sherman EJ , Ho AL , Fagin J , et al. Combination of dabrafenib (DAB) and lapatinib (LAP) for the treatment of BRAF mutant thyroid cancer. J Clin Oncol. 2018;36(15 Suppl):S6087.

